# Incarcerated Ventral Hernia Requiring Emergent Repair in an Adult With Down Syndrome: Diagnostic and Postoperative Challenges in an Underrepresented Population

**DOI:** 10.7759/cureus.104459

**Published:** 2026-02-28

**Authors:** Zuhayr Khan, Mahsum Jafri, Constantino G Lambroussis

**Affiliations:** 1 General Medicine, Lake Erie College of Osteopathic Medicine, Elmira, USA; 2 Internal Medicine, Lake Erie College of Osteopathic Medicine, Elmira, USA; 3 Osteopathic Medicine/Family Medicine, Lake Erie College of Osteopathic Medicine, Elmira, USA

**Keywords:** alzheimer's disease, down syndrome, hernia, incarcerated ventral hernia, strangulated ventral hernia

## Abstract

The manifestation of ventral hernias is quite common, with an increased risk of progressing to incarceration/strangulation, which imposes the need for emergent surgery. Patients with Down syndrome (DS) present unique anatomical, physiological, and neurodevelopmental characteristics that may complicate diagnosis and postoperative management. In general, DS has been well studied for perioperative risk primarily with pediatric and cardiac populations, yet emergency general surgical outcomes in adults remain poorly studied. In our case report, a 59-year-old woman with DS and Alzheimer’s disease underwent emergent robotic ventral hernia repair for an incarcerated ventral hernia. Her postoperative course was complicated by several features, including obstipation, urinary retention with bilateral hydronephrosis, and the development of bilateral segmental and subsegmental pulmonary emboli on postoperative day four. Interventions for these complications, such as a Foley catheter insertion for urinary retention, led to additional complications. Diagnosis and management were primarily guided by physical examination and cross-sectional imaging, given the patient’s limited ability to provide a reliable clinical history. Our case report highlights the challenges of standard postoperative care in adults with DS and also stresses the importance of personalized monitoring, diagnostic imaging, and multidisciplinary approaches, especially for individuals with complex disease processes.

## Introduction

Ventral hernias can often arise spontaneously and may require surgical intervention when they become a life-threatening emergency, such as when incarceration or strangulation occurs. Due to the emergent nature of this procedure, and as described in our case presentation, it is often associated with higher morbidity than an elective repair, especially in patients with complex medical or neurodevelopmental conditions, such as our patient with Down syndrome (DS). DS, or trisomy 21, is the most common chromosomal abnormality, occurring in 1:700-1:800 live births. DS is associated with many factors that can lead to perioperative and postoperative complications such as hypotonia, connective tissue abnormalities, immune dysregulation, airway anomalies, altered pain perception, and variable communication abilities [1]. In addition, there have been increased postoperative complications that have been documented across multiple surgical subspecialties in patients with DS such as orthopedic, otolaryngologic, and spinal procedures [2,3]. Perioperative management in DS requires particular attention to airway anatomy, medical comorbidities such as obstructive sleep apnea, general hypotonia, and cardiopulmonary comorbidities, which may significantly influence anesthetic risk and postoperative recovery [4].

Gastrointestinal pathology and genitourinary pathology are especially prevalent in DS, as individuals with DS exhibit higher rates of congenital and functional gastrointestinal abnormalities such as malrotation, diaphragmatic hernias, gastroesophageal reflux disease, chronic constipation, and feeding difficulties. The unfortunate reality is that many of these disease processes present atypically or are diagnosed late [5]. Hernia-related pathology in DS is prone to diagnostic delay and recurrence, as seen with Morgagni and other anterior diaphragmatic hernias disproportionately affecting this population and frequently identified only after multiple healthcare provider visits/encounters [6,7]. Adult cases of hernia repair in DS have been reported to require complex surgical and perioperative management due to associated comorbidities and atypical presentation [8]. Although these vulnerabilities are recognized and are known to have increased effect in this patient population, yet there still remains a lack of literature describing emergency general surgery and postoperative outcomes in adults with DS. Our case highlights the diagnostic and postoperative challenges encountered following surgical intervention, more specifically emergent ventral hernia repair, in an adult with DS and emphasizes the importance of individualized postoperative monitoring and early diagnostic imaging.

## Case presentation

**Figure 1:**
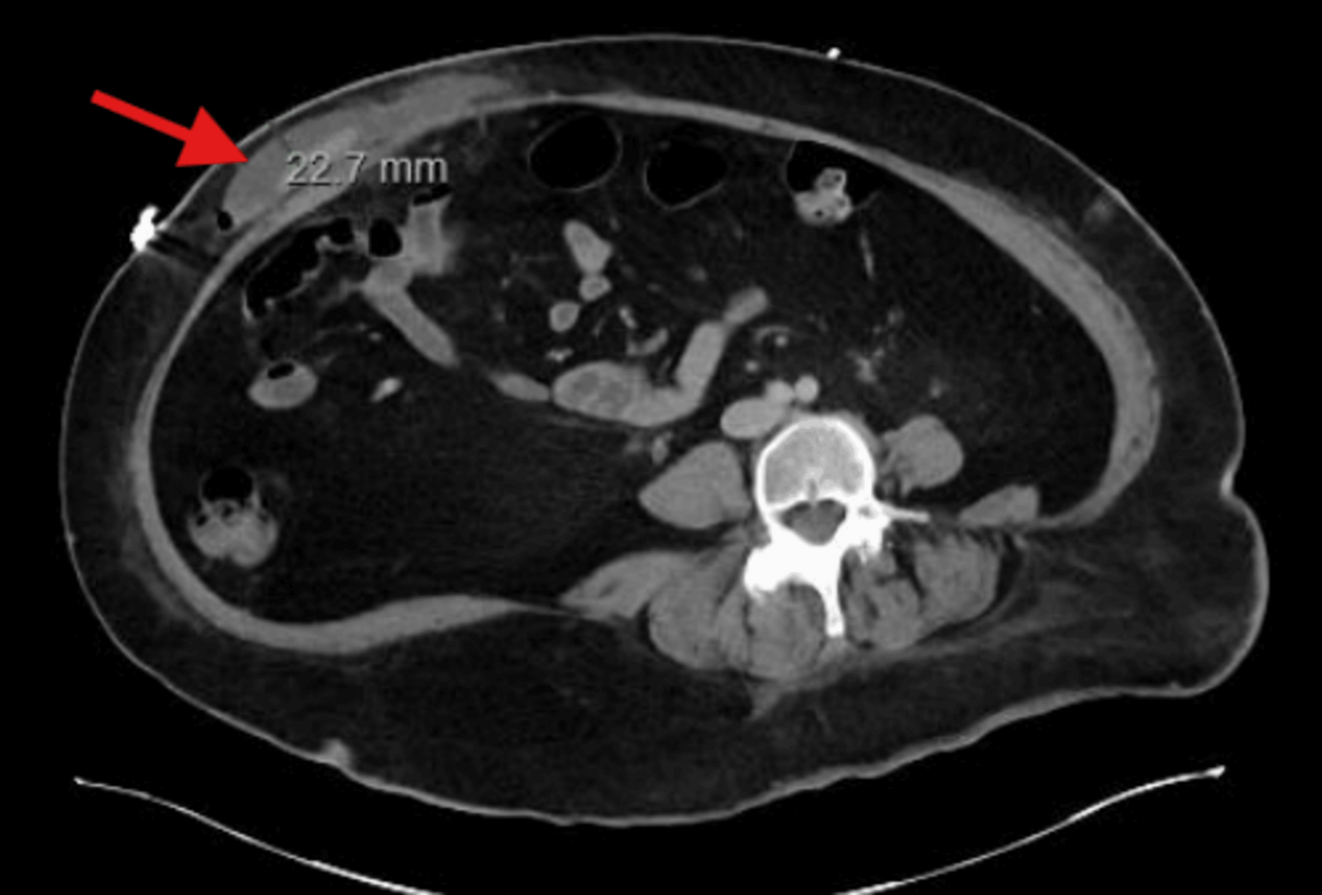
CT of abdomen and pelvis with contrast showing an approximately 2 cm mixed density fluid collection with small internal foci of air involving the right anterior lateral abdominal wall that is marked with a red arrow

**Figure 2:**
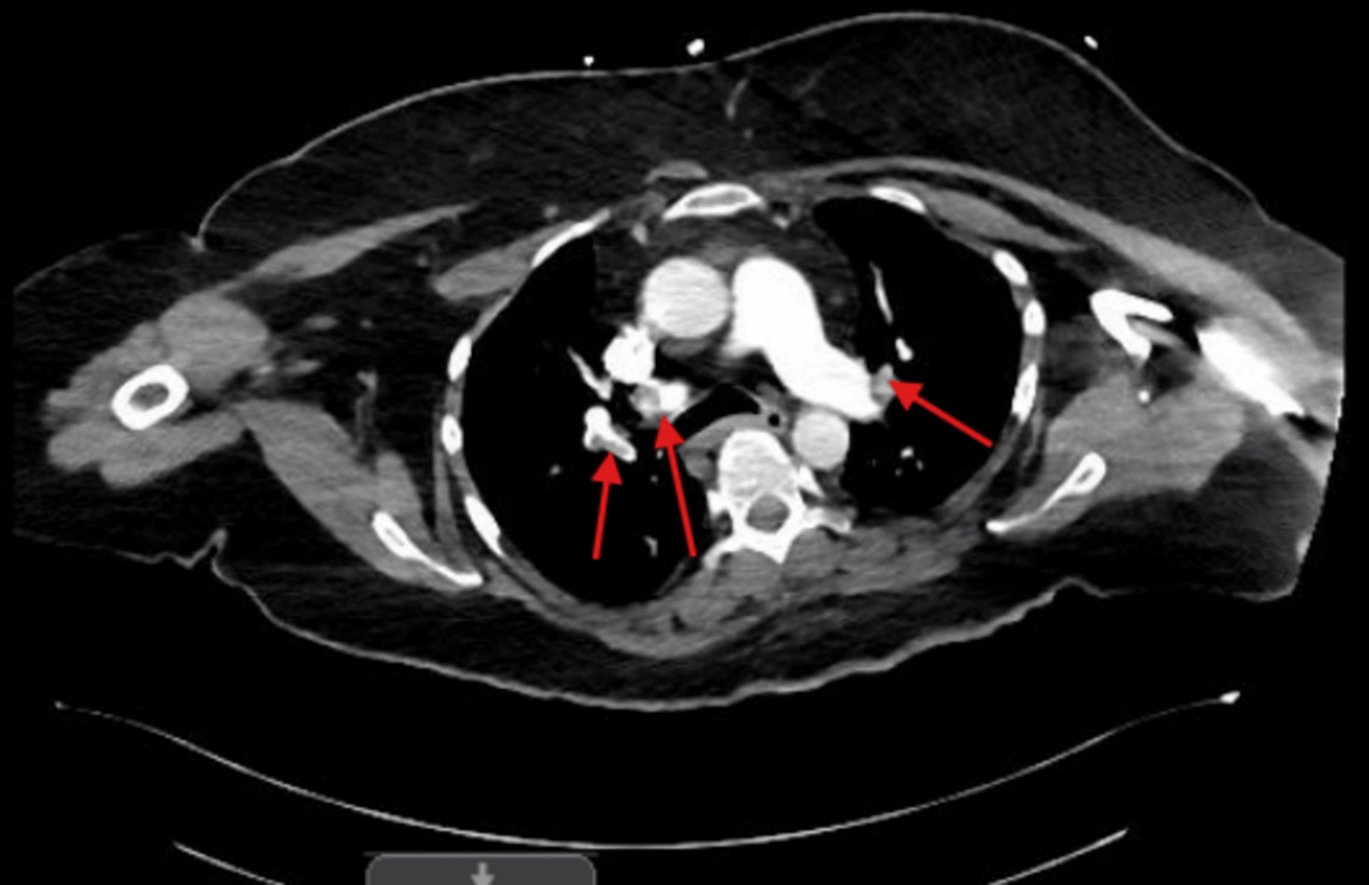
CT chest angiogram revealing bilateral segmental and subsegmental pulmonary emboli in the upper-mid lobes of the lung, which are depicted by the red arrows.

**Figure 3:**
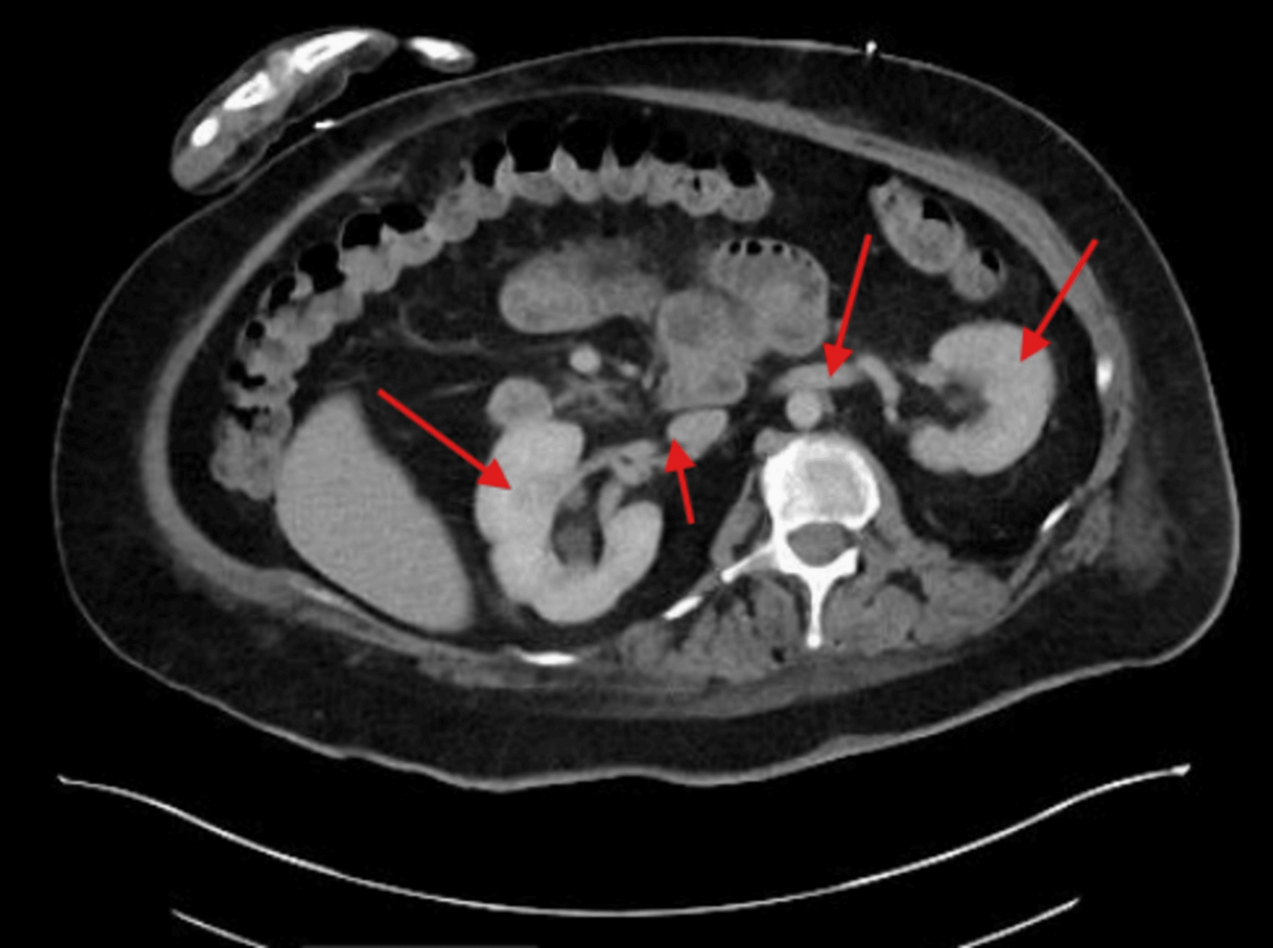
CT of abdomen and pelvis with IV contrast depicts bilateral hydronephrosis without an obstructing stone. Red arrows point out the bilateral hydronephrosis.

A 59-year-old woman with a history of DS, Alzheimer’s disease, and other comorbidities initially presented with a non-reducible ventral abdominal wall mass with associated tenderness, concerning for an incarcerated ventral hernia. Physical exam of the abdomen and pelvis demonstrated a 5 cm incarcerated ventral hernia. Given the risk of progression to strangulation, the patient was taken emergently to the operating room. The patient underwent robotic ventral hernia repair with 9 cm mesh placement under general anesthesia. From an anesthesiology perspective, airway management in this patient was approached by maintaining neutral alignment while minimizing neck extension. In addition, manual in-line stabilization and intubation with video laryngoscopy were carefully used as needed. The procedure was completed without any intraoperative complications. In Figure 1, a postoperative computed tomography (CT) scan was done, which showed an approximately 2 cm mixed-density fluid collection with small internal foci of air involving the right anterior lateral abdominal wall, which can be expected following recent surgery and is suspected to likely be a small hematoma, seroma, or abscess. Postoperatively, clinical assessment was limited by difficulty in pain localization and communication. The patient was immediately admitted in medicine service postoperatively and instructed bedrest for four hours, and any pain was managed by the medicine team. The patient exhibited delayed return of bowel function and only passed flatus, necessitating close multidisciplinary monitoring. Given concern for potential postoperative complications, a low threshold (high-risk patient, given the history) was maintained for repeat imaging and objective assessment. On postoperative day four following hernia repair, the patient presented to the emergency department with altered mental status. The patient’s SpO2 was in the high 70s and low 80s. A CT angiogram of the chest was done, which demonstrated bilateral segmental and subsegmental pulmonary emboli, which can be seen in Figure 2. A chest X-ray was also done, which demonstrated diffuse ground-glass opacification of the lungs and cardiomegaly. The patient’s family also noted that our patient had difficulty urinating at home, and a CT of the abdomen and pelvis confirmed bilateral hydronephrosis, as seen in Figure 3, due to post-op urinary retention, and thus a Foley catheter was placed. The Foley catheter was planned to remain in place for at least one month.

Eight days later, the Foley catheter was prematurely removed, and the patient presented for an urgent follow-up appointment with abdominal pain, abdominal distention, and hematuria following self-removal of her Foley catheter. An ultrasound was done at this appointment, and it revealed mild bilateral hydronephrosis and thickened bladder walls. The post-void residual volume was 229 mL.

## Discussion

This case illustrates the limitations of standard postoperative care pathways when applied to adults with DS undergoing emergency abdominal surgery. While ventral hernia repair is routinely performed with predictable outcomes in the general population, patients with DS possess several physiological and neurodevelopmental factors that may obscure early recognition of postoperative complications. Altered pain perception and communication barriers are well-documented in DS and have a complicated postoperative assessment [1,9]. In our case, difficulty with pain localization and symptom reporting limited reliance on subjective assessment, necessitating objective evaluation and close multidisciplinary monitoring. These challenges increased the risk of delayed identification of complications such as ileus, infection, or thromboembolic disease, as was the case with our patient. Gastrointestinal and genitourinary dysfunction further complicates postoperative evaluation in DS. In the DS patient population, it could be difficult to identify if GI/GU dysfunction is present. 

A study demonstrated a high prevalence of gastrointestinal anomalies and functional disorders in individuals with DS, many of which present with nonspecific or subtle symptoms [5]. In our case, delayed return of bowel function and postoperative urinary retention with bilateral hydronephrosis illustrate how baseline functional abnormalities can mask an evolving pathology. Prior studies have shown that hernia-related pathology in DS is frequently diagnosed late and often requires advanced imaging for clarification [6,7]. Reports of complex hernia repair in adults with DS further emphasize the need for heightened perioperative vigilance in this population [8].

Another factor to put into elevated consideration is the thromboembolic risk, as it represents a particularly high-stakes postoperative concern in adults with DS. A fatal case of postoperative venous thromboembolism following epigastric hernia repair in an adult with DS has been reported, highlighting the potentially catastrophic consequences of delayed recognition [10]. In our case, she developed bilateral segmental and subsegmental pulmonary emboli on postoperative day four (as seen in Figure 2) and presented primarily with altered mental status and hypoxia rather than classic cardiopulmonary complaints. This atypical presentation further emphasizes the need for heightened suspicion and objective evaluation.

From an imaging and interventional radiology perspective, our case reinforces the critical role of early cross-sectional imaging when clinical examination and symptom reporting are unreliable or confounding. CT imaging was essential in this case for identifying postoperative abdominal findings, such as confirming pulmonary emboli with CT angiography and diagnosing bilateral hydronephrosis. Prior literature has emphasized the importance of imaging in DS patients with atypical gastrointestinal and hernia-related presentations, particularly when diagnosis is delayed or physical examination is inconclusive [6,7]. Therefore, early integration of diagnostic imaging and image-guided evaluation may reduce delays in diagnosis and improve outcomes in this susceptible population.

## Conclusions

Surgical procedures such as emergency ventral hernia repair in adults with DS present challenges that extend beyond the operating room. Adults with DS exhibit distinct clinical characteristics, including altered pain perception, communication limitations, gastrointestinal and genitourinary dysfunction, perioperative anesthetic complexities, and an increased risk of thromboembolic events, which may obscure or delay the recognition of postoperative complications when perioperative management is not appropriately tailored to this population. This case report emphasizes the importance of individualized postoperative monitoring, having a low threshold for diagnostic imaging, and considering multidisciplinary collaboration to improve outcomes in adults with DS undergoing emergency surgery.
